# *Entamoeba histolytica* Contains an Occludin-Like Protein That Can Alter Colonic Epithelial Barrier Function

**DOI:** 10.1371/journal.pone.0073339

**Published:** 2013-09-13

**Authors:** Michael Goplen, Manigandan Lejeune, Steve Cornick, France Moreau, Kris Chadee

**Affiliations:** Gastrointestinal Research Group, Department of Microbiology, Immunology and Infectious Diseases, Snyder Institute for Chronic Diseases, Faculty of Medicine, University of Calgary, Calgary, Alberta, Canada; University of Chicago, United States of America

## Abstract

The exact mechanism by which *Entamoeba histolytica* disrupts the human colonic epithelium and invades the mucosa has yet to be clearly elucidated. *E. histolytica* produces a diverse array of putative virulent factors such as glycosidase, cysteine proteinases and amebapore that can modulate and/or disrupt epithelial barrier functions. However, it is currently thought that *E. histolytica* produces numerous other molecules and strategies to disrupt colonic mucosal defenses. In this study, we document a putative mechanism whereby the parasite alters the integrity of human epithelium by expressing a cognate tight junction protein of the host. We detected this protein as “occludin-like” as revealed by immunoblotting and immunoprecipitation studies and visualization by confocal microscopy using antibodies highly specific for human occludin. We propose that *E. histolytica* occludin-like protein might displace mucosal epithelial occludin-occludin tight junction interactions resulting in epithelial disruption analogous to sub mucosal human dendritic cells sampling luminal contents. These results indicate that *E. histolytica* occludin is a putative virulent component that can play a role in the pathogenesis of intestinal amebiasis.

## Introduction

*Entamoeba histolytica* is a protozoan parasite that colonizes the mucus barrier in the colon and can lead to amebiasis [1]. The parasite infects over 50 million people annually resulting in 100,000 deaths, causing a significant impact on global health [2]. Several factors responsible for the pathogenesis of the disease have been elucidated in recent years including the parasite surface adhesion molecule Gal/GalNAc lectin (Gal-lectin) and secreted and membrane bound cysteine proteases and lipophosphopeptidoglycans [3], [4], [5]. Many of the defined virulence factors disrupt colonic mucosal barrier integrity, specifically tight junction proteins that result in diarrhea or more serious amebic dysentery [6]. However, the current thought is there are many other virulence factors that are yet to be defined that can modulate epithelial barrier functions.

Tight junctions (TJ) are dynamic structures located on the apical region of the epithelial paracellular space and maintain contiguity of the epithelial lining. The TJ is composed of a mosaic of proteins that form a semi-permeable paracellular diffusion barrier [7]. These dynamic junctions consist of 40 different proteins that include occludin, claudins, zona occludens, junctional adhesion molecules (JAMS) and the coxsackievirus and adenovirus receptor (CAR) [7]. In a recent study [8], we demonstrated that prostaglandin E_2_ secreted by *E. histolytica* disrupted TJ by selectively affecting one of its components, claudin-4. This change increased paracellular permeability, leading to a leaky gut [8]. Thus, altering the TJ barrier is one strategy that ameba utilizes to disrupt the colonic barrier to gain access to sub mucosal space. The exact mechanism how the parasite does this is unknown.

In a recent report [9], human dendritic cells (DC) were shown to employ a unique mechanism of penetrating human colonic epithelial TJ. DC was reported to express one of the TJ proteins, occludin on its plasma membrane. Occludin, a transmembrane protein has two extra cellular loops that normally interact with its counterparts on adjacent epithelial cells to seal the paracellular space of the TJ [10]. Indeed, DC expresses occludin to selectively open the paracellular space and sample the colonic lumen by competing for epithelial occludin-occludin interactions to create new DC-epithelial occludin-occludin interaction and ultimately, a passage through paracellular space. These observations form the basis of the idea that pathogens could utilize an occludin-like protein to pathologically disrupt the TJ barrier. Consistent with this concept, a recent study [11] showed that using synthetic peptides designed to parallel the extracellular regions of occludin, impaired epithelial barrier integrity. Since *E. histolytica* trophozoites interact intimately with the colonic mucosa and can alter barrier functions, we hypothesize that the parasite might express a TJ protein and use a strategy similar to DC to disrupt the epithelium. In this study, we detected that *E. histolytica* expresses a protein that has similarities with human occludin, including its extracellular loop and demonstrate a role in altering barrier functions. To our knowledge, this is the first report of *E. histolytica* expressing a protein similar to a host TJ protein that can impair human colonic barrier integrity.

## Materials and Methods

### Antibodies and reagents

Primary antibodies to occludin (C-terminal) were obtained from Invitrogen (Carlsbad, CA). Antibodies to the extracellular loop peptide of occludin and the corresponding blocking peptides were purchased from Santa Cruz Biotechnology (Santa Cruz, CA). FITC conjugated secondary antibody was from Abcam (Cambridge, MA). All other chemical were procured from Sigma-Aldrich (St. Louis, MO).

### Preparation of SAP

Soluble Amebic Proteins (SAP) were prepared and collected as described previously [12]. Briefly, a highly virulent strain of *E. histolytica* HM1-IMSS (sub-passage in gerbil liver), were cultured until mid log phase in TYI-S-33 medium containing heat inactivated 20% adult bovine serum. Trophozoites were washed three times in ice-cold Hanks' balanced salt solution and were subjected to three cycles of freeze-thaw. The proteins were centrifuged for 10 min (5000×g) and the supernatant containing SAP were quantified by Bradford's protein assay, aliquoted and stored at −80°C until further use.

### Immunoblotting

SAP or SAP depleted of the “occludin-like” proteins was mixed with equal volume of 1× sample buffer, boiled for 10 min and used for Western blotting. Approximately 20 µg of total protein was loaded per well of 12% SDS-PAGE and electrophoresis was performed. Proteins were transferred onto nitrocellulose membranes (Bio-Rad, Hercules, CA) followed by blocking with 5% skim milk powder in TBS-T (20 mmol/L Tris-HCl, pH 7.5, 500 mmol/L NaCl, 0.1% Tween 20) for 1 hr at room temperature. Membranes were incubated with appropriate primary antibodies in 1% skim milk-TBS-T at 4°C, overnight. Blots were washed three times with TBS-T and then incubated in horseradish peroxidase-conjugated secondary antibodies in 1% skim milk-TBS-T for 2 hr at room temperature. The blots were washed with TBS-T and were developed using Immobilon Western chemiluminescent HRP substrate (Millipore, Billerica, MA) according to the manufacturer's instructions. T84 cells grown to confluence on a 6 well plates were centrifuged at 1250×g for 5 min at 4°C. The pellet was resuspended in 400 µl of ice-cold cell lysis buffer (50 mmol/L Tris-HCl, 140 mmol/L EDTA, 30 mmol/L sodium pyrophosphate, 50 mmol/L sodium fluoride and protease inhibitor cocktail tablet) containing 1% Triton X-100. Protein content was assayed using Bradford's method. T84 cells were used as control.

### Immunoprecipitation

SAP or T84 cellular lysates (300 µg of protein in 600 µl of lysis buffer) were incubated for 4 hr at 4°C under constant rocking, with rabbit anti occludin antibodies. The lysates were further incubated overnight with 50 µl of protein G-Agarose beads. Pellets were collected by brief centrifugation at 4000×g, washed four times with 400 µl of buffer, and subjected to SDS-PAGE. Immunoblotting protocol was followed as indicated before.

### Immunofluorescence confocal microscopy

*E. histolytica* grown in glass culture tubes were chilled on ice for 10 min. After inverting to detach the trophozoites from the glass wall, cells were transferred to a falcon tube and centrifuged at 4°C at 200×g for 5 min. The pellet was washed 3 times with ice cold sterile PBS. After a final wash, the pellet was fixed with 400 µl of 3.5% paraformaldehyde at 4°C for 15 min. After washing with PBS-2% Tween 20 (PBS-T), the trophozoites were permeabilized using 0.35% Triton X-100 in PBS for 5 min and blocked with 5% BSA in PBS-2% Tween 20 (PBS-T) for 30 min at room temperature. Trophozoites were incubated for 1 hr in 1∶100 dilution of occludin antibody at room temperature. After two washes in PBS-T, trophozoites were co-incubated with fluorescein isothiocyanate (FITC)-conjugated secondary antibodies (1∶200 dilutions) for 30 min at room temperature. After two washes with PBS-T, slides were treated with 4′, 6-diamidino-2-phenylindole (DAPI; Invitrogen) to stain the nucleus. The trophozoites were then placed on a clean glass slides and allowed to dry. Before it completely dried, coverslip was mounted on the glass slide using Vectashield (Vector Laboratories, Burlingame, CA). Slides were examined using a FluoView FV1000 confocal immunofluorescence microscope (Olympus). Immunofluorescence signals were captured in individual en face (*xy* axes) planes throughout the cellular *z-axis* at 0.35 µm intervals. Z-planes obtained were stacked and analyzed using Volocity software version 6.1 (PerkinElmer, Waltham, MA). In experiments involving trophozoites mounted on transwell plates; the parasites were first allowed to settle on transwell and fixed directly with methanol.

### T84 cell culture and transwell preparation

T84 human colonic cells (ATCC, Manassas, VA) were maintained in Dulbecco's modified Eagle's medium with Ham's F-12 supplemented with 10% fetal bovine serum, 100 units/mL penicillin, 100 g/mL streptomycin sulfate and 20 mmol/L HEPES. T84 cells grown to confluence on a 6 well plates were used for experiments involving immunoblot and immunoprecipitation. For trans epithelial resistance (TER) experiments, colonic cells were prepared as described previously [8]. Briefly, T84 cells grown to 100% confluence on polyethylene membrane inserts (12-mm diameter, 0.4 µm pore size; catalog no. 3460, Corning Costar Transwell; Corning Life Sciences, Corning, NY) were used. TER was measured using a Millicell-ERS apparatus (Millipore, Bedford, MA). When a stable resistance of 2000 Ω·cm^2^ was reached, the medium from the apical compartment was replaced with medium containing SAP or SAP depleted of occludin-like proteins and TER was measured. Changes in TER were normalized to that of the baseline resistance (resistance at the starting time point) and represented as percentage of the control over a desired time course. The permeability of the TJ was measured by studying the apical to basal translocation [8] of FITC-dextran (2–4 KDa) at 5, 15, 30 and 60 min following the addition or not of SAP.

### Statistical analysis

Data were analyzed by two-way analysis of variance for comparison between groups using GraphPad Prism version 4.0 (Graph-Pad Software, San Diego, CA). Data are reported as means ± SD of three independent experiments.

## Results

### Immunoblot detection of the occludin-like protein in *E. histolytica*

**Figure 1:**
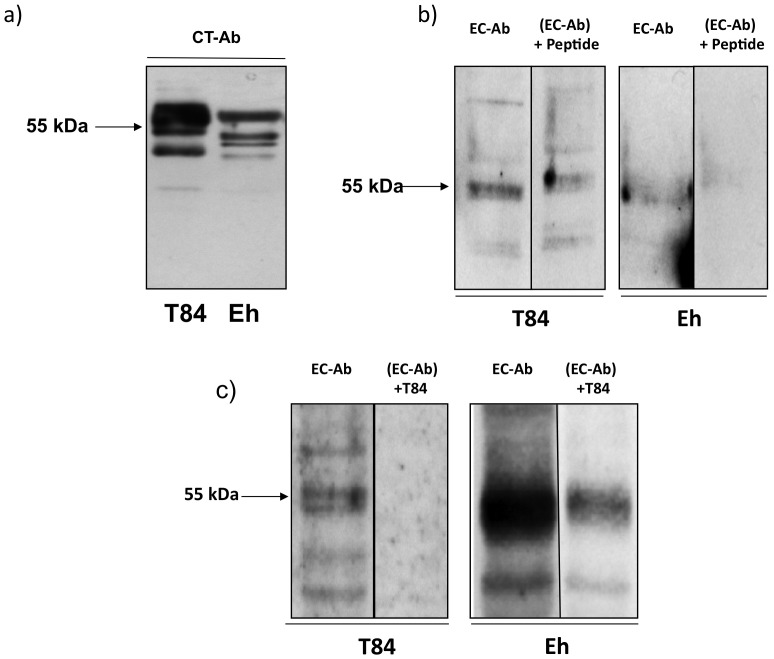
Identification of the “occludin-like” protein in *E. histolytica*. (a) Immunoblot showing the 55 KDa protein of *E. histolytica* (Eh) probed with an antibody that recognizes the C-terminus of human occludin (CT-Ab). T84 human colonic epithelial cells were used as a control. Note the multiple phosphorylated occludin proteins (>55 KDa) observed in human T84 cells are absent in Eh. (b) Immunoblot using an antibody that recognizes the extra cellular loops of mouse occludin (EC-Ab) detected the 55 KDa occludin-like protein in Eh SAP. The EC-Ab blocking peptide (15-fold excess) inhibited EC-Ab binding of the Eh 55 kDa protein by 83% and in T84 control cells by 89%. (c) Similar to (b) above, T84 lysates used at 10-fold excess inhibited binding of the Eh occludin-like protein by 80% and 100% in T84 cells. Each experiment was repeated three times with similar results and representative blots are shown.

To determine whether *E. histolytica* expresses a TJ protein, whole soluble amebic proteins (SAP) were immunoblotted using a human occludin antibody that detect the 55 kDa C-terminus domain. As shown in Figure 1a, the 55 kDa protein in ameba (Eh) had similar immunoreactivity comparable to occludin expressed in T84 human colonic epithelial cells. This protein was designated the putative “occludin-like” protein of *E. histolytica*. The molecular weight of human occludin ranges from 55–72 kDa as a result of multiple phosphorylation states. Therefore, the higher bands observed in T84 cells are phosphorylated occludin proteins which are not observed in SAP. However, the 55 kDa band was of interest since it is within the molecular weight range of human occludin (Fig. 1a). Interestingly, the “occludin-like” protein of *E. histolytica* was also detected using a mouse occludin antibody that recognizes the extra cellular loop (Fig. 1b). As depicted in Figure 1b, the occludin antibody detected a 55-kDa protein of *E. histolytica* that was inhibited 83% by the addition of a blocking peptide (+Peptide). Specificity for the antibody was also shown using T84 cellular lysates rich in occludin (Fig. 1a) that specifically inhibited (100%) binding to the different forms of T84 occludin and the *E. histolytica* “occludin-like” protein (80%; Fig. 1c). These results suggest that *E. histolytica* has a 55 kDa protein that shares similarity to the extracellular loops of occludin.

### Immunoprecipitation of the “occludin-like” protein in *E. histolytica*

**Figure 2:**
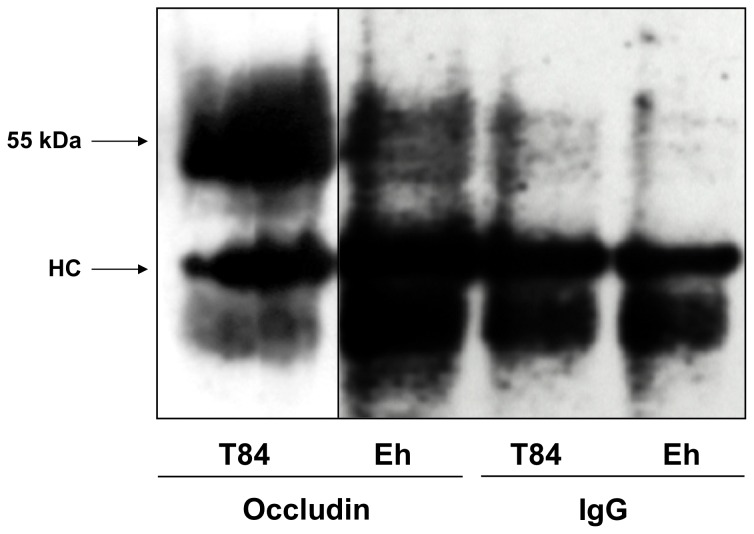
Human occludin C-terminus antibody immunoprecipitated a 55 kDa protein from *E. histolytica*. Immunoprecipitation of T84 human colonic cells and Eh cellular lysates were done using a rabbit occludin antibody and subsequently immunoblotted with a mouse occludin antibody. Note the presence of 55“occludin-like” protein in Eh. The heavy chain (HC) is shown on the blots. IgG was used as isotype control. This experiment was repeated three times with similar results and representative blots are shown.

To address the nature of the “occludin-like” protein present in *E. histolytica*, immunoprecipitation (IP) studies were performed on SAP using a human occludin C-terminus antibody raised in rabbit and subsequently immunoblotted with a C-terminus antibody raised in mouse. As shown in Figure 2, a prominent 55 kDa protein was pulled-down with the occludin antibody from SAP that was similar to that observed in the previous experiment (Figure 1a). These results demonstrate and further confirm the presence of a major single “occludin-like” protein in *E. histolytica*. The dense 55 KDa band in T84 cells may represent different phosphorylated forms of occludin. The Ig heavy chain (HC) is clearly evident using the occludin and the IgG control antibody.

### Visualization of the occludin-like protein in *E. histolytica* by confocal microscopy

**Figure 3:**
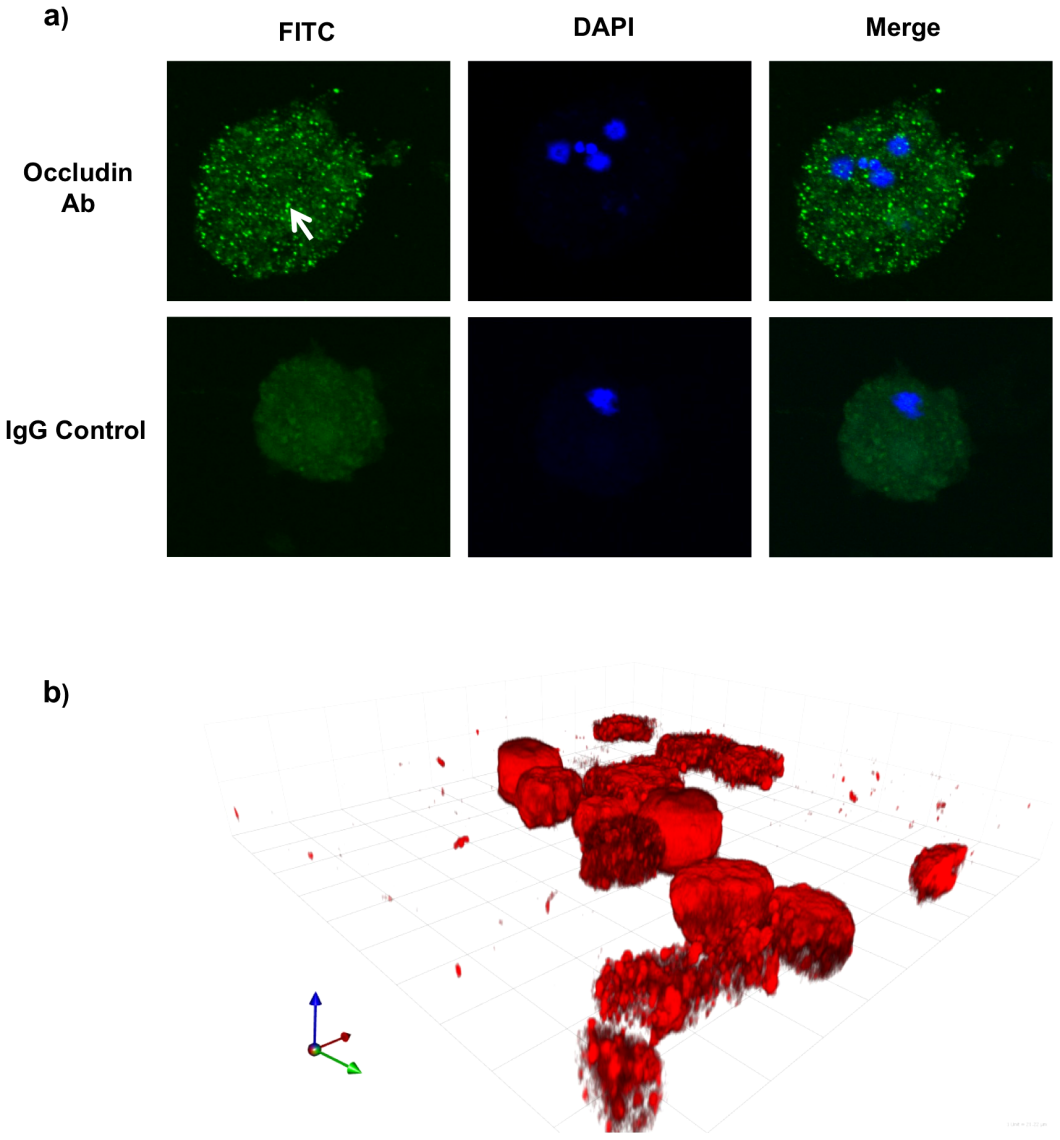
Immunofluorescent confocal microscopy of *E. histolytica*. (a) Trophozoites were incubated with or without human C-terminus occludin antibody (Ab) followed by FITC tagged species-specific secondary antibody. DAPI was used to counter stain nuclei. Arrow points to intense green staining for the occludin-like proteins. Note that the intense green staining is absent in the IgG controls. (b) 3D reconstruction of Z-stacks of slices along xy planes of ameba attached on transwell membranes throughout the cellular z-axis at 0.35 µ intervals of trophozoites probed for human occludin C-terminus protein. The red fluorescence tag indicates occludin. An isotype control IgG showed no binding (data not shown). This experiment was repeated twice with similar results.

To determine the cellular localization and distribution of the “occludin-like” protein in *E. histolytica* trophozoites, confocal immunofluorescence microscopy was performed. As shown in Figure 3a, the “occludin-like” protein in ameba stained as diffuse intense green punctuate proteins (arrow) throughout the cytoplasm. A similar distribution of the “occludin-like” proteins was seen when ameba was allowed to attach to transwell membranes and the proteins analyzed by 3D reconstruction of Z-stacks (Figure 3b). The images were over exposed to visualize if there were any regional localization of the occludin-like protein in ameba. These results confirm the presence of the “occludin-like” protein in *E. histolytica* throughout the cytoplasm and it is not distributed along the leading edge of the pseudopodia.

### The occludin-like protein of *E. histolytica* impairs human colonic epithelial barrier integrity

**Figure 4:**
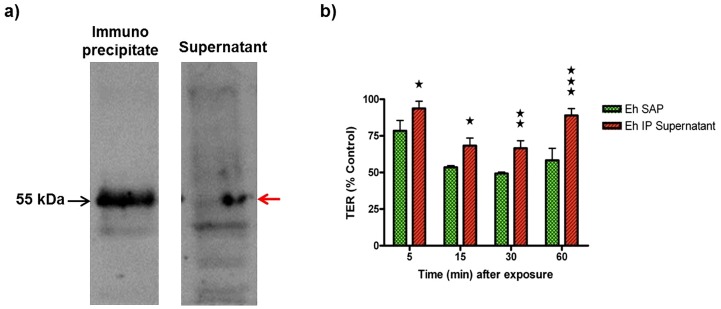
*E. histolytica* occludin-like protein impairs T84 epithelial barrier integrity. (a) Depletion of 55 KDa proteins from SAP by immunoprecipitation using a human occludin C-terminus antibody. Note that the 55 kDa band in the supernatant following immunoprecipitation was decreased (red arrow). (b) T84 monolayers on transwells were treated with either 20 µg soluble amebic protein (Eh SAP) or 20 µg of SAP depleted of occludin-like protein (Eh IP Supernatant) and compared to the control (T84 monolayer treated with HBSS). The un-normalized baseline mean (of three replicates) TER at the start of the experiment were 4276 Ω·cm^2^ for Eh SAP, 3840 Ω·cm^2^ for Eh IP Supernatant and 3993 Ω·cm^2^ for controls. Control isotype IgG had no effect on TER. Stars represent statistical significance between the Eh SAP and Eh IP supernatant (*P<0.05; ** P<0.01; ***P<0.001). This experiment was repeated twice with similar results.

As the synthetic peptide corresponding to the extra cellular loop of occludin was shown to decrease epithelial paracellular barrier integrity [11], we postulated that the putative 55 kDa “occludin-like” protein of *E. histolytica*, detected by both the C-terminus and the extracellular loop antibody of occludin, can function similarly to that of the synthetic extracellular loop peptide. To test this notion, experiments were performed to quantify the effect of SAP with or without the putative “occludin-like” protein on human colonic epithelial barrier integrity. To do this, SAP was depleted of the 55 kDa “occludin-like” protein by immunoprecipitation using the human occludin C-terminus antibody as shown in Figure 4a, which demonstrates that the putative protein was depleted in the supernatant by 60%. We next tested the effect of SAP (Eh SAP) with or without the “occludin-like” protein (Eh IP supernatant) by adding to the apical surface of a contiguous monolayer of T84 human colonic epithelium grown on transwell plates. This was done by recording changes in trans epithelial resistance (TER) that measures the integrity of the epithelial monolayer as previously described [8]. As shown in Figure 4b, freshly prepared SAP significantly decreased TER at all the time points measured (5, 15, 30 and 60 minutes after exposure), reaching a maximum between 15 and 30 minutes (@51% decrease in TER compared to control monolayer). However, SAP depleted of the “occludin-like” protein (red histogram bars) did not markedly decrease the TER. For example, at 30 minutes TER was decreased only 33% as compared to a 51% decrease by SAP. The same trend was noted for all the time points measured where the difference in TER between SAP and SAP-depleted of the “occludin-like” protein was statistically significant. Moreover, SAP did not alter the permeability to low molecular weight (3–4 kDa) FITC-dextran suggesting that the paracellular pathway was not severely affected. These results confirm that the decrease in TER caused by SAP was due, in part, to the occludin-like protein.

## Discussion

In this study we demonstrate the presence of a 55 kDa “occludin-like” protein in *E. histolytica* that can disrupt TER in human colonic epithelial cells. The identity of the protein was confirmed using highly specific antibodies raised against the C-terminus and the extracellular loop of occludin. The presence of the “occludin-like” protein in *E. histolytica* was confirmed by immunoblotting, immunoprecipitation and immunofluorescence confocal microscopy. Human occludin is a 55 kDa protein that has multiple phosphorylation states, which causes wide variations in its observed molecular weight [10]. However, higher molecular weight bands were not observed in *E. histolytica*, suggesting that the protein may have a different pattern of phosphorylation or may be a distinct protein that only shares partial sequence similarity with that of occludin.

Our results suggest that the “occludin-like” protein has sequence similarities with the extracellular loop of mammalian occludin. An antibody that was raised against the extracellular loop of occludin detected a 55 kDa protein in SAP. To confirm specificity, we used a blocking peptide against the extracellular loop antibody and T84 cellular lysates enriched in occludin to compete out the binding. As predicted, both the blocking peptide and T84 cellular lysates eliminated the extracellular loop antibody ability to detect the 55 Da *E. histolytica* protein. This demonstrates that the “occludin-like” protein of *E. histolytica* has domains similar to the extracellular loop of occludin. Proteomic analysis using domain enhanced basic local alignment search tools (DELTA BLAST; NCBI) identified a previously uncharacterized hypothetical protein (XP_652128.1) of 579 amino acids in the *E. histolytica* proteome which has homology to the C-terminal domain of human occludin. Previous reports have implicated the C-terminal domain of occludin as having a coiled-coil domain (D426-E469), which affords the interaction with other TJ proteins, particularly zonula occludens [12]. Using an algorithm previously described that can predict coiled-coil domains (COILS Server), the presence of a coiled-coil domain was confirmed for both human occludin (Approx 425–470 aa) and the “occludin-like” protein (XP_65218.1; 350–480 aa) [13]. The “occludin-like” protein in ameba was present in the cytoplasm as diffuse punctuate proteins and did not polarize to the leading ends of pseudopodia when trophozoites were attached to transwell membranes. While the surface of the pseudopodia is predicted to contact the TJ of colonic epithelia to alter TER, we cannot rule out the possibility that the “occludin-like” protein can also be secreted onto the epithelia, but this is only speculation. The two extracellular loops of occludin have been shown to be integral in creating and maintaining epithelial barrier integrity [10]. Moreover, extraneous peptides corresponding to certain amino acids in the extracellular loops disrupted epithelial barrier integrity [10], [11]. Thus, the 55 kDa “occludin-like” protein may have amino acid sequence similar to the extracellular loop of human occludin and may disrupt human epithelial barrier integrity by displacing human occludin interactions. The evidence for this is that the “occludin-like” protein of *E. histolytica* was shown to disrupt human colonic epithelial TJ barrier integrity. We elucidated that SAP significantly decreased TER when compared to SAP depleted of the occludin-like protein. Thus, any difference in the decrease of TER was due to the “occludin-like protein” that specifically competed for occludin-occludin interaction at the TJ similar to that observed using synthetic peptides [10], [11].

In summary, this study describes the presence of a 55 kDa “occludin-like” protein in *E. histolytica* using various specific mammalian occludin antibodies and confocal microscopy. As the “occludin-like” protein specifically decreased TER on human colonic epithelial monolayer, it may contribute to the pathogenesis of intestinal amebiasis.
